# Efficacy of intermittent theta-burst stimulation for substance use disorders: a systematic review and meta-analysis of randomized sham-controlled trials

**DOI:** 10.3389/fneur.2026.1866761

**Published:** 2026-08-05

**Authors:** Guohua Jiang, Jiliang Kang, Rui Jiang, Qiwu Dong

**Affiliations:** Ningbo Rehabilitation Hospital, Ningbo, China

**Keywords:** abstinence, alcohol, craving, intermittent theta-burst stimulation, meta-analysis, methamphetamine, nicotine, substance use disorder

## Abstract

**Objective:**

To evaluate the efficacy of intermittent theta-burst stimulation (iTBS) for substance use disorders (SUDs), with craving reduction as the primary outcome and abstinence as the secondary outcome, and to examine whether treatment effects differed by substance type.

**Methods:**

PubMed, Embase, the Cochrane Library, Web of Science, and PEDro were searched from inception to March 17, 2026 for randomized sham-controlled trials of prefrontal iTBS in individuals with SUDs. Standardized mean differences (SMDs) were calculated for craving outcomes and odds ratios (ORs) for abstinence. Random-effects models, substance-specific subgroup analyses, and leave-one-out sensitivity analyses were used as appropriate.

**Results:**

Nine randomized sham-controlled trials involving 556 participants were included. Eight trials (*n* = 512) contributed continuous craving data. Active iTBS was associated with a reduction in craving relative to sham stimulation (SMD = −0.65, 95% CI −1.08 to −0.21; *p* = 0.003), although heterogeneity was substantial (*I*^2^ = 81%). Subgroup analyses showed a larger effect in methamphetamine use disorder (five trials, *n* = 311; SMD = −1.03, 95% CI −1.29 to −0.77; *I*^2^ = 11%) and no detectable effect in nicotine use disorder (three trials, *n* = 201; SMD = 0.01, 95% CI -0.28 to 0.30; *I*^2^ = 3%). Three trials involving nicotine or alcohol use disorder contributed abstinence data; the pooled estimate favored active iTBS (OR = 2.19, 95% CI 1.09 to 4.39), but the evidence base was small and follow-up was short.

**Conclusion:**

Current evidence suggests that prefrontal iTBS may reduce craving in methamphetamine use disorder, but the overall evidence remains substance-specific and should not be interpreted as demonstrating efficacy across all SUDs. No benefit was detected for nicotine craving. The preliminary abstinence finding requires confirmation in larger trials with longer follow-up before conclusions can be drawn regarding sustained abstinence or relapse prevention.

**Systematic review registration:**

PROSPERO, CRD420261363708.

## 1 Introduction

Substance use disorders (SUDs) are chronic, relapsing conditions associated with substantial health and social burdens (1). Their development and persistence involve maladaptive neuroplasticity within mesocorticolimbic and executive-control networks, including impaired prefrontal regulation of reward-related and cue-reactive processes (2, 3). Although pharmacological and behavioral interventions are effective for some patients, relapse remains common and treatment options are particularly limited for stimulant use disorders (4). These limitations have encouraged investigation of circuit-based interventions that directly modulate neural systems implicated in craving and inhibitory control.

Non-invasive brain stimulation has emerged as a potential adjunctive treatment for SUDs (5). Repetitive transcranial magnetic stimulation (rTMS) can alter cortical excitability and influence functionally connected reward networks (6). Conventional high-frequency prefrontal rTMS is the excitatory magnetic stimulation approach most often studied in addiction and has shown potential for reducing craving; however, standard protocols usually require longer session times, repeated clinic visits, and substantial clinical resources, and treatment effects vary across substances and protocols (7–9). These limitations have encouraged interest in more time-efficient patterned protocols.

Intermittent theta-burst stimulation (iTBS) is a patterned form of rTMS that delivers bursts at theta frequency and can induce facilitatory, long-term-potentiation-like changes in cortical excitability (10, 11). Compared with conventional high-frequency rTMS, a standard iTBS session delivers stimulation within a substantially shorter time, which may improve feasibility, throughput, and adherence in clinical populations (12, 13). Nevertheless, greater practical efficiency should not be assumed to imply equivalent or broader efficacy across different SUDs, and the therapeutic effects of iTBS therefore require evaluation separately from conventional high-frequency rTMS protocols.

The prefrontal cortex was selected as the focus of this review because it contributes to inhibitory control, salience processing, decision-making, and regulation of substance-related cues, and it is the principal cortical target used in randomized iTBS trials for addiction (14). Nevertheless, the neurobiology of methamphetamine, nicotine, and alcohol use disorders is not identical, and differences in receptor systems, cue processing, habitual behavior, and network dysfunction may modify treatment response (15–17). Therefore, a pooled estimate across all substances may obscure clinically relevant variation.

Previous reviews have commonly combined iTBS with conventional rTMS, deep TMS, or other neuromodulation approaches, limiting conclusions specific to iTBS (14). In addition, recently published randomized trials have expanded the available evidence. In the current evidence base, methamphetamine and nicotine were the only substance categories with sufficient comparable continuous craving data for subgroup pooling, whereas one alcohol use disorder trial contributed to the secondary abstinence analysis.

Accordingly, the primary objective of this systematic review and meta-analysis was to determine whether active prefrontal iTBS reduces craving more effectively than sham stimulation in individuals with SUDs. Secondary objectives were to assess abstinence outcomes, compare craving effects between methamphetamine and nicotine use disorders, examine the robustness of pooled estimates, and identify limitations relevant to future substance-specific neuromodulation trials.

## 2 Methods

### 2.1 Protocol and registration

This review was reported in accordance with the Preferred Reporting Items for Systematic Reviews and Meta-Analyses (PRISMA) 2020 statement (18). The final database search was completed on March 17, 2026. The protocol was registered in PROSPERO on April 8, 2026 (CRD420261363708), after the review had commenced and before initial manuscript submission on April 27, 2026. At registration, the review was ongoing rather than prospectively uninitiated; exact day-level dates for each internal screening, extraction, and synthesis step had not been prospectively logged. Accordingly, the registration is not described as prospective. Because the review used data from previously published studies and involved no direct participation of human subjects, ethics approval was not required.

### 2.2 Search strategy

PubMed, Embase, the Cochrane Library, Web of Science, and PEDro were searched from inception to March 17, 2026. The search combined controlled vocabulary and free-text terms for intermittent theta-burst stimulation or iTBS; broad substance-use concepts, including substance use disorder and addiction, together with methamphetamine, nicotine, tobacco, smoking, alcohol, cocaine, opioid, and cannabis terms; and randomized, sham, placebo, or controlled-trial concepts. Eligibility was not restricted to the substances named in the search description, and reference lists of relevant reviews and eligible studies were screened manually. The search was restricted to peer-reviewed English-language reports. The complete database-specific search strategies, including syntax and field tags, are provided in Appendix 1.

### 2.3 Study selection

Records were imported into EndNote X9 and duplicates were removed. Two reviewers independently screened titles and abstracts and subsequently assessed potentially eligible full texts. Disagreements were resolved by discussion with a third reviewer. Studies were eligible if they: (1) used a randomized sham-controlled design; (2) enrolled participants with a formally diagnosed SUD, without restriction to a single substance; (3) applied active iTBS to a prefrontal target; and (4) reported extractable craving or abstinence data. The prefrontal restriction was specified to reduce target-related clinical heterogeneity and because prefrontal circuits are central to executive control and cue regulation in addiction. Studies were excluded if they used non-randomized or open-label designs, applied unequal co-interventions between groups, or did not provide sufficient data for quantitative synthesis. Substance-specific subgroup analysis was conducted only when at least two studies reported comparable outcome data; this criterion was met for methamphetamine and nicotine craving, whereas the alcohol trial contributed only to the abstinence analysis.

### 2.4 Data extraction and management

Two investigators independently extracted data using a standardized form. Extracted variables included author and year, country, sample size, substance type, participant characteristics, active and sham stimulation procedures, cortical target, intensity, pulse number, treatment schedule, follow-up duration, outcome measures, and principal findings. A third investigator verified all entries against the source reports. When the standard deviation of a change score was unavailable, it was estimated using a correlation coefficient of 0.5. The characteristics of the included trials are summarized in Table 1.

**Table 1:** Characteristics of the included randomized sham-controlled trials.

Author, year	Substance	Country	E/C (n)	Age, years (mean ± SD)	Active procedure	Sham procedure	Target and iTBS parameters	Outcomes / Assessment	Principal findings
Chen et al. (23)	Methamphetamine	China	30/19	E: 29.7 ± 4.7 C: 30.7 ± 6.7	iTBS plus standard rehabilitation.	Same schedule; coil rotated 180° away from the scalp, plus standard rehabilitation.	L-DLPFC (Beam F3); 20 sessions/4 weeks; 900 pulses/session; 100% RMT; 50-Hz triplets at 5 Hz; 2 s on/8 s off.	Craving weekly from baseline to week 4; Addiction Stroop and EEG pre/post treatment.	Active iTBS produced a greater craving reduction and reduced errors for methamphetamine-related words; N1/P3 and beta-band responses were also modulated.
Dieler et al. (24)	Nicotine	Germany	38/36	E: 46.7 ± 10.1 C: 46.3 ± 9.5	Four iTBS sessions added to a 3-week group CBT program.	Same CBT program; 60% MT with the coil tilted 45°.	R-DLPFC (F4); 4 sessions over 2 weeks; 600 pulses/session; active intensity 80% MT; 50-Hz triplets at 5 Hz.	Craving pre/post treatment; continuous abstinence assessed at 3, 6, and 12 months.	No between-group difference in craving. Abstinence was higher after active iTBS at 3 months, but not at 6 or 12 months.
Durazzo et al. (25)	Alcohol	USA	22/22	E: 50.6 ± 14.1 C: 51.3 ± 14.1	Active side of an A/P coil plus residential treatment as usual.	Placebo side of the A/P coil with time-synchronized forehead electrical stimulation, plus the same residential treatment.	L-DLPFC (Beam F3); 20 sessions in ≤14 days (2-3/day); 1,200 pulses/session; 100–110% aMT; 50-Hz triplets at 5 Hz.	Percent heavy-drinking days, continuous abstinence, and alcohol consumption during 6-month follow-up.	Active iTBS produced a larger reduction in heavy-drinking days. Continuous abstinence occurred in 13/22 versus 9/22 participants; among those who resumed drinking, abstinence duration was longer and alcohol consumption was lower.
Mikellides et al. (26)	Nicotine	Cyprus	59/30	E: 44.7 ± 13.8* C: 47.4 ± 12.7	Active aiTBS with neutral cues (*n* = 29) or smoking-related cues (*n* = 30).	Placebo side of a Cool-B65 A/P coil with smoking-related cues (*n* = 30).	L-DLPFC (Beam F3); 20 sessions/5 days (4/day; 30-min intervals); 600 pulses/session; 100% RMT; 50-Hz triplets at 5 Hz.	Cigarette use, exhaled CO, nicotine dependence, craving, and stress; end of treatment and 1-week follow-up.	Cigarette consumption, nicotine dependence, and craving improved similarly in all groups; active aiTBS was not superior to sham.
Ren et al. (27)	Methamphetamine	China	24/25	E: 33.7 ± 6.1 C: 35.5 ± 6.6	Active iTBS without a differential co-intervention.	Same schedule and acoustic sensation; coil rotated 90° away from the scalp.	L-DLPFC (F3); 20 sessions/4 weeks (5/week); 600 pulses/session; 100% RMT; 50-Hz triplets at 5 Hz; 2 s on/8 s off.	Cue-induced craving, impulsivity, anxiety, and depression at baseline and week 4.	Active iTBS yielded greater reductions in craving, impulsivity, anxiety, and depression; improvement in depression and impulsivity was associated with craving response.
Su et al. (28)	Methamphetamine	China	70/56	E: 31.9 ± 6.1 C: 31.4 ± 6.6	Active iTBS within residential rehabilitation.	Same parameters with the coil rotated 180° away from the scalp.	L-DLPFC (F3); 20 sessions/4 weeks; 900 pulses/session; 100% RMT; 50-Hz triplets at 5 Hz; 2 s on/8 s off.	Craving assessed weekly; cognition and sleep after 4 weeks; relapse monitored after rehabilitation.	Active iTBS significantly reduced cue-induced craving and improved cognition and sleep; relapse data were limited by few events.
Su et al. (29)	Methamphetamine	China	25/25	E: 32.4 ± 6.6 C: 31.9 ± 6.2	Active iTBS plus regular rehabilitation.	Same parameters with the coil rotated 180° away from the scalp, plus regular rehabilitation.	L-DLPFC (Beam F3); 20 sessions/4 weeks; 900 pulses/session; 100% RMT; 50-Hz triplets at 5 Hz; 2 s on/8 s off.	Cue-induced craving pre/post treatment; resting-state fMRI within 1 week after the 4-week intervention.	Active iTBS reduced craving and altered frontoparietal and insular connectivity; increased DLPFC-IPL connectivity correlated with craving reduction.
Addicott et al. (17)	Nicotine	USA	25/13	E: 56 ± 12 C: 54 ± 15	Active iTBS with smoking-cue priming and smoking-cessation education.	Integrated sham coil with forehead electrical stimulation, plus identical cue priming and education.	mPFC (AFz); 28 sessions over 14 visits (2/visit; 20-30-min interval); 600 pulses/session; 110% RMT; 50-Hz triplets at 5 Hz.	Cigarettes/day, cotinine-verified abstinence, craving, withdrawal, and mood through four weekly follow-up visits.	Both groups reduced cigarette use and craving, with no significant active-sham differences; verified abstinence was 16% versus 15%.
Chen et al. (22)	Methamphetamine	China	18/19	E: 37.7 ± 4.3 C: 35.0 ± 4.8	L-DLPFC iTBS plus treatment as usual.	Sham TBS with a magnetically shielded placebo coil, plus treatment as usual.	L-DLPFC (active arm); 10 sessions/2 weeks; 900 pulses/session; active iTBS at 100% RMT; 50-Hz triplets at 5 Hz; 2 s on/8 s off.	Cue-induced craving and response rate; emotion, withdrawal, sleep, and cognition after 2 weeks.	L-DLPFC iTBS significantly reduced craving and increased the responder rate compared with sham stimulation.

Characteristics of the included randomized sham-controlled trials, including substance type, sample size, participant age, active and sham procedures, cortical target and iTBS parameters, outcome measures, assessment schedule, and principal findings. Values are mean ± SD. E/C denotes the active iTBS and sham groups included in the meta-analysis. *For Mikellides et al., the two active arms were pooled for the meta-analysis; the pooled age was calculated from the reported arm-level data. Abbreviations: A/P, active/placebo; aiTBS, accelerated intermittent theta-burst stimulation; aMT, active motor threshold; CBT, cognitive behavioral therapy; CO, carbon monoxide; DLPFC, dorsolateral prefrontal cortex; EEG, electroencephalography; fMRI, functional magnetic resonance imaging; IPL, inferior parietal lobule; L-DLPFC, left dorsolateral prefrontal cortex; mPFC, medial prefrontal cortex; MT, motor threshold; R-DLPFC, right dorsolateral prefrontal cortex; RMT, resting motor threshold.

### 2.5 Quality assessment

Risk of bias was assessed independently by two reviewers using the original Cochrane risk-of-bias tool implemented in Review Manager 5.4 (19). This tool was retained because it had been prespecified in our extraction framework and applied consistently across all included trials before manuscript preparation. We acknowledge that RoB 2 is currently recommended for randomized trials; however, retrospective conversion to RoB 2 without consistent access to trial protocols and prespecified statistical analysis plans could introduce additional judgment variability. To maintain consistency and transparency, we reported domain-level judgments from the original tool and explicitly considered unclear allocation concealment and selective reporting when interpreting the evidence.

### 2.6 Data analysis

Quantitative analyses were performed in Review Manager 5.4. For continuous craving outcomes, standardized mean differences (SMDs) with 95% confidence intervals (CIs) were calculated. For dichotomous abstinence outcomes, odds ratios (ORs) with 95% CIs were calculated. Heterogeneity was evaluated using Cochran’s Q test and the *I*^2^ statistic (20). A random-effects model was used for craving because clinical and methodological heterogeneity was anticipated across substances and protocols (21). A fixed-effect model was used for the abstinence analysis because only three studies contributed and no statistical heterogeneity was detected. Substance-specific subgroup analyses were undertaken when at least two comparable trials were available. Robustness of the craving estimate was examined by sequentially omitting each study. Because fewer than 10 studies contributed to each analysis, funnel plots and Egger’s tests were not performed; publication bias was therefore considered qualitatively in the Discussion and Limitations, with attention to the small evidence base and the predominance of positive findings in methamphetamine trials. Statistical significance was defined as a two-sided *p* < 0.05.

## 3 Results

### 3.1 Search results

**Figure 1:**
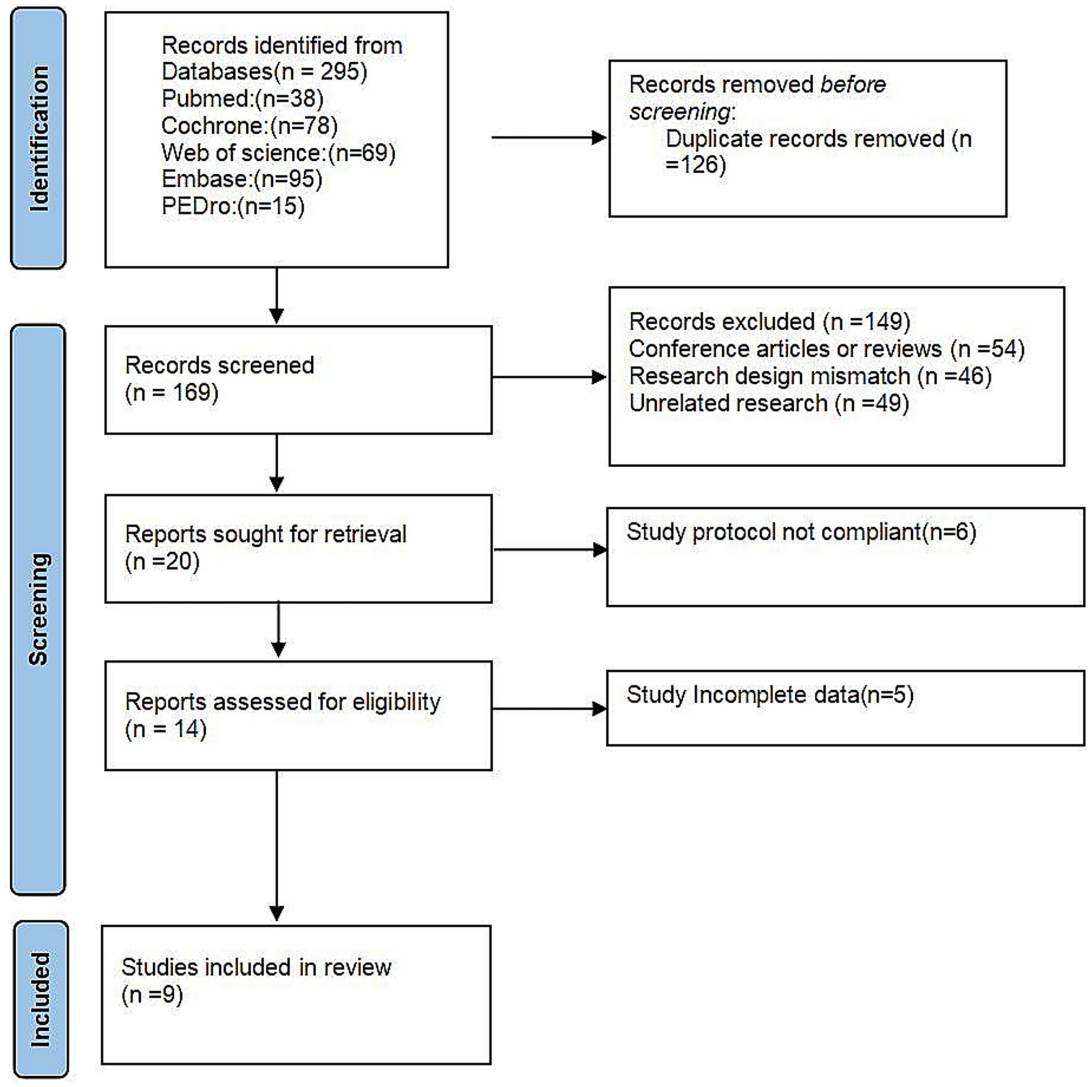
PRISMA 2020 flow diagram showing study identification, screening, eligibility assessment, and inclusion.

The database search identified 295 records. After removal of 126 duplicates, 169 records underwent title and abstract screening, and 149 were excluded because of an ineligible design, publication type, or topic. Twenty reports were sought for full-text retrieval. Six were excluded because the intervention did not meet the prespecified iTBS criteria, leaving 14 reports for eligibility assessment; five of these did not provide extractable outcomes. Nine randomized sham-controlled trials were included in the qualitative synthesis (17, 22–29). Eight trials contributed craving data and three contributed abstinence data. The selection process is shown in Figure 1.

### 3.2 Study characteristics

The nine included trials, published between 2014 and 2025, enrolled 556 participants in total. Eight trials involving 512 participants contributed to the craving meta-analysis; the additional trial in alcohol use disorder contributed to the abstinence analysis. Five trials investigated methamphetamine use disorder (*n* = 311), three investigated nicotine use disorder (*n* = 201), and one investigated alcohol use disorder (*n* = 44). Five studies were conducted in China (22, 23, 27–29), two in the United States (17, 25), and one each in Germany (24) and Cyprus (26). Seven trials targeted the left dorsolateral prefrontal cortex (22, 23, 25–29), one targeted the right dorsolateral prefrontal cortex (24), and one targeted the medial prefrontal cortex (17). Table 1 provides the sample characteristics, active and sham procedures, stimulation parameters, outcomes, follow-up, and principal findings for each study.

### 3.3 Risk of bias

**Figure 2:**
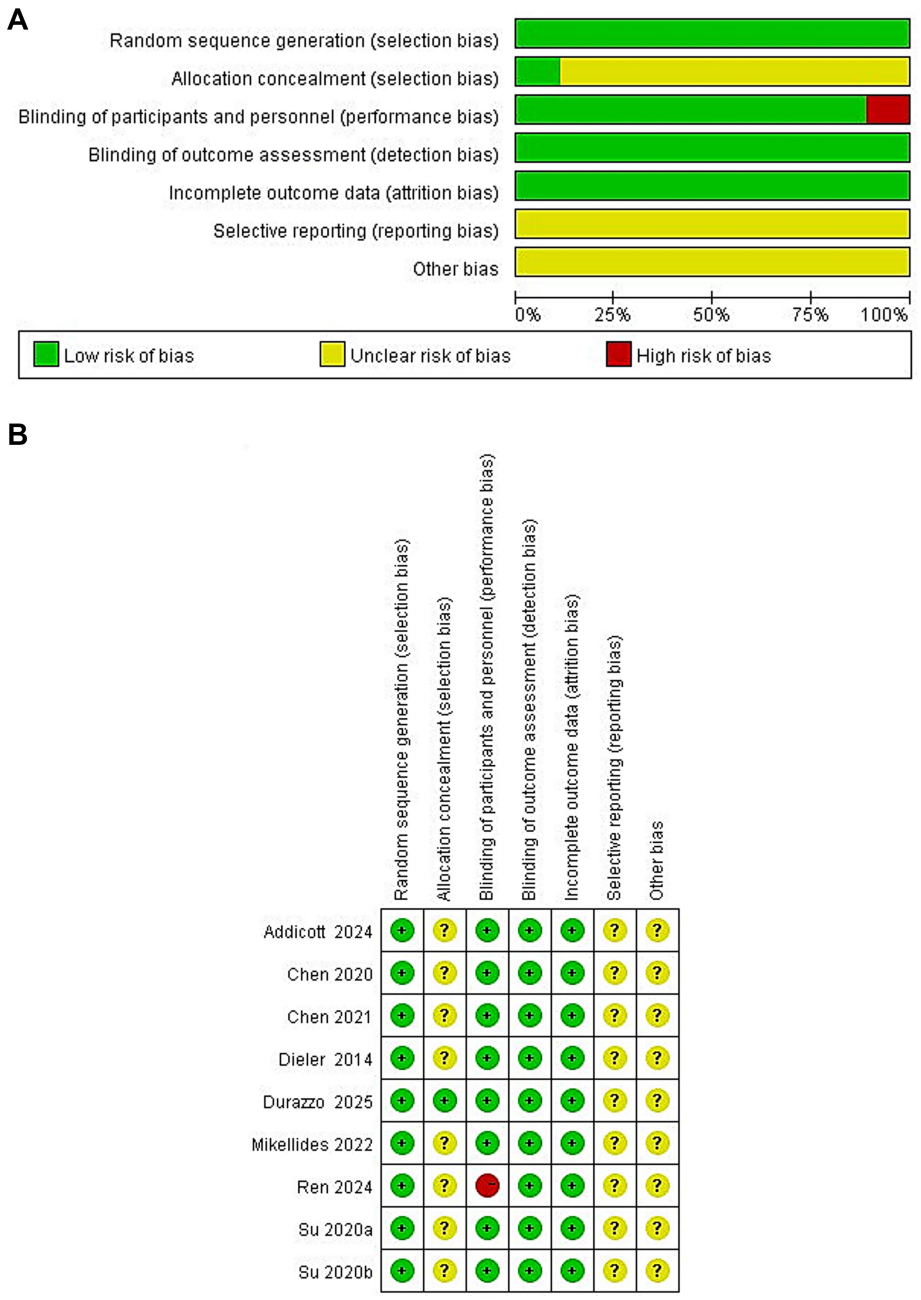
Risk-of-bias assessment of the included randomized trials: **(A)** Distribution of judgments across bias domains; **(B)** Study-level risk-of-bias summary.

The risk-of-bias assessments are presented in Figures 2A,B. All nine studies reported randomized allocation and were judged to have a low risk of bias for sequence generation. Allocation concealment was described adequately in one trial (25), while the remaining studies were judged to have an unclear risk because reporting was insufficient. Eight trials reported blinding of participants and personnel; one study (27) was judged to have a high risk of performance bias. All trials were judged to have a low risk of detection and attrition bias. Selective reporting and other bias were frequently rated as unclear because protocols or sufficiently detailed prespecified analyses were unavailable.

### 3.4 Quantitative synthesis of craving

#### 3.4.1 Overall effect

**Figure 3:**
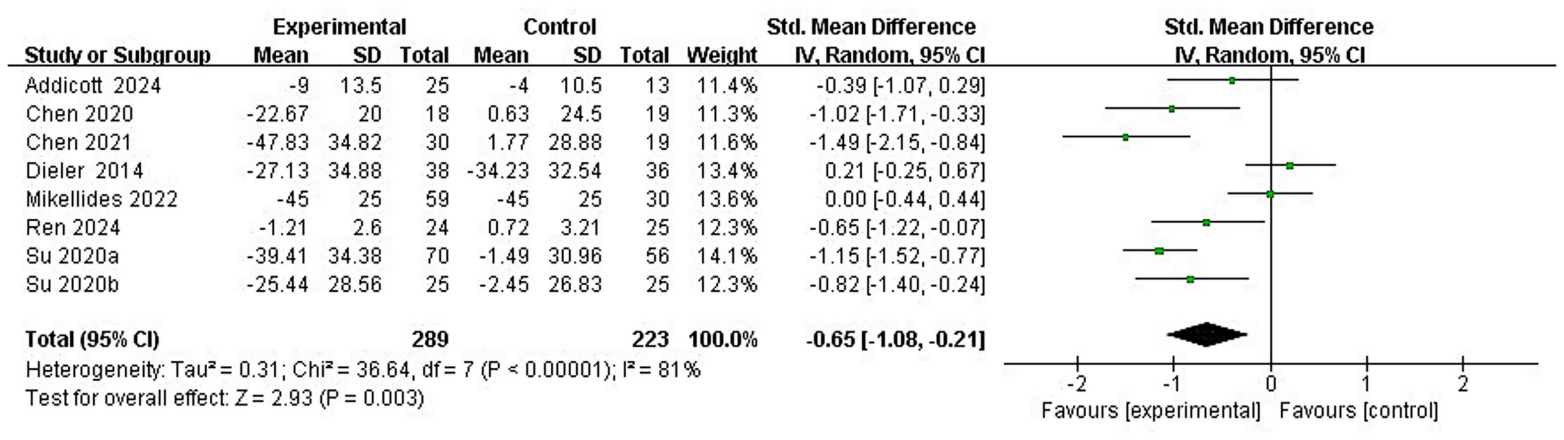
Forest plot comparing active iTBS with sham stimulation for the overall craving outcome.

Eight studies involving 512 participants provided continuous craving data (17, 22–24, 26–29). Active iTBS was associated with lower craving scores than sham stimulation (SMD = −0.65, 95% CI −1.08 to −0.21; Z = 2.93; *p* = 0.003; Figure 3). Statistical heterogeneity was substantial (*I*^2^ = 81%; *p* < 0.00001), indicating that the overall estimate should be interpreted cautiously. Sequential omission of individual studies did not materially change the direction or statistical significance of the pooled estimate.

#### 3.4.2 Subgroup analysis by substance type

**Figure 4:**
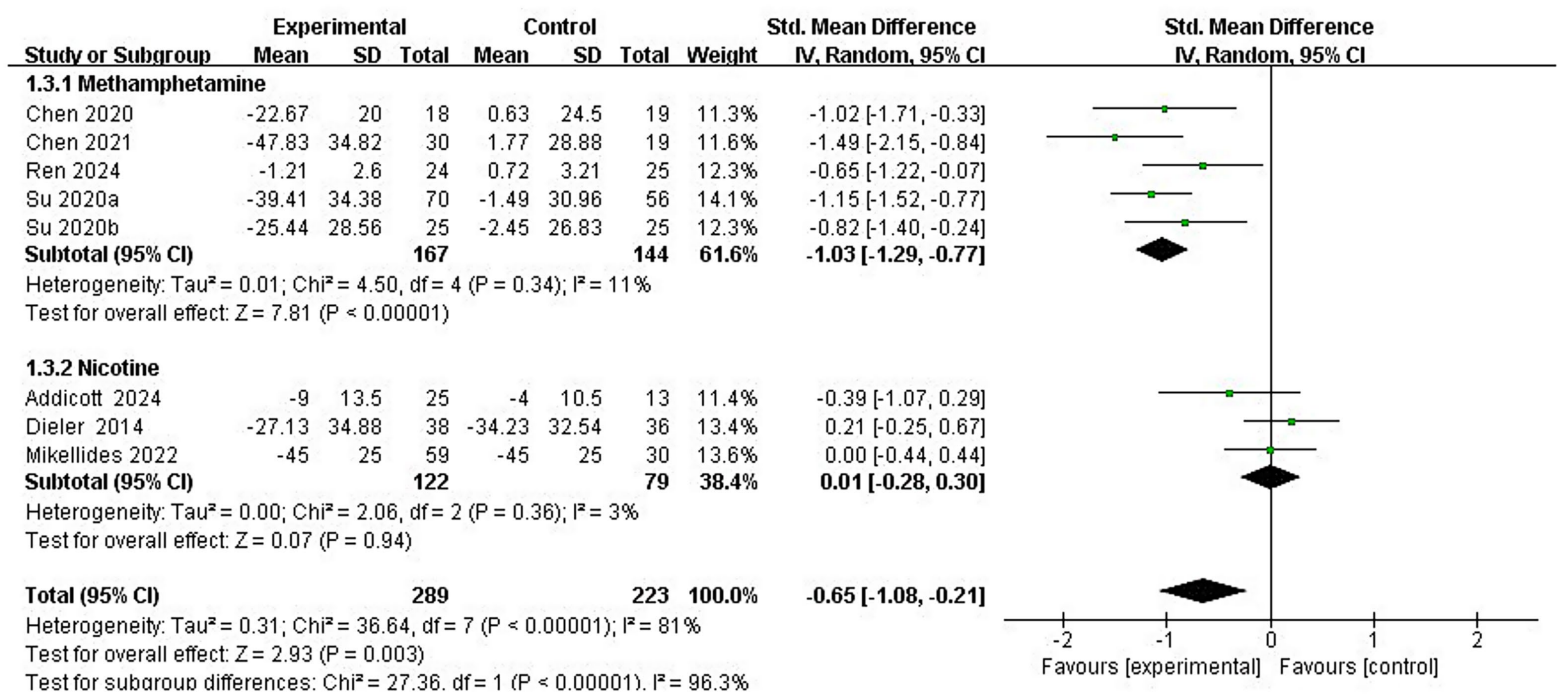
Forest plot of the substance-specific subgroup analysis for craving in methamphetamine and nicotine use disorders.

Subgroup analyses were performed for methamphetamine and nicotine because these were the only substance categories represented by at least two trials with comparable continuous craving outcomes (Figure 4).

Methamphetamine use disorder: Five studies involving 311 participants were included (22, 23, 27–29). Active iTBS was associated with a reduction in craving relative to sham stimulation (SMD = −1.03, 95% CI −1.29 to −0.77; *p* < 0.00001), with low within-subgroup heterogeneity (*I*^2^ = 11%; *p* = 0.34).

Nicotine use disorder: Three studies involving 201 participants were included (17, 24, 26). No difference in craving was detected between active and sham iTBS (SMD = 0.01, 95% CI −0.28 to 0.30; *p* = 0.94), and heterogeneity was low (*I*^2^ = 3%).

The test for subgroup differences was statistically significant (*p* < 0.00001; *I*^2^ = 96.3%). This finding indicates that the pooled estimates differed between the two subgroups, but it does not establish that substance type alone caused the difference because the trials also varied in stimulation target, treatment schedule, sample characteristics, and outcome measurement.

### 3.5 Abstinence outcomes

**Figure 5:**
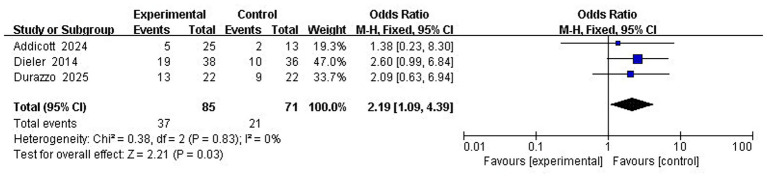
Forest plot comparing active iTBS with sham stimulation for abstinence outcomes. The analysis included two trials in nicotine use disorder and one trial in alcohol use disorder.

Three trials involving nicotine or alcohol use disorder evaluated abstinence as a dichotomous outcome (17, 24, 25). Active iTBS was associated with higher odds of abstinence than sham stimulation (OR = 2.19, 95% CI 1.09 to 4.39; Z = 2.21; *p* = 0.03; Figure 5), with no observed statistical heterogeneity (*I*^2^ = 0%; *p* = 0.83). Because this analysis included only three small trials, combined different substances, and was based on short follow-up intervals, the result should be regarded as preliminary and should not be interpreted as evidence of sustained relapse prevention.

## 4 Discussion

This meta-analysis included nine randomized sham-controlled trials with 556 participants. Eight trials contributed craving data, and the pooled estimate favored active iTBS, although heterogeneity was substantial. The overall effect was driven primarily by the methamphetamine subgroup, in which the effect estimate was larger and heterogeneity was low. In contrast, no reduction in nicotine craving was detected. Three nicotine or alcohol trials contributed abstinence data, producing a statistically significant pooled OR; however, the small evidence base and short follow-up preclude firm conclusions regarding sustained abstinence.

### 4.1 Craving outcomes and substance-specific variation

The methamphetamine subgroup showed the clearest evidence of benefit. This finding is consistent with prefrontal-striatal dysfunction, dopaminergic dysregulation, impaired inhibitory control, altered cortical and network organization, and planning deficits reported in methamphetamine use disorder (30–35). Excitatory stimulation of the dorsolateral prefrontal cortex may influence these processes through changes in cortical excitability and connected frontostriatal networks, with preliminary evidence also suggesting effects on executive and working-memory function (36–40). Nevertheless, the included trials measured clinical craving rather than neural mechanisms directly; mechanistic explanations should therefore be considered biologically plausible hypotheses rather than demonstrated mediators of the pooled effect.

The null estimate for nicotine craving should not be interpreted as definitive evidence that iTBS is ineffective for tobacco use disorder. Nicotine dependence involves distributed networks that include prefrontal, insular, salience, and interoceptive systems, and the included nicotine trials differed in target selection, sham technology, treatment intensity, and concurrent behavioral support (17, 24, 26, 41–43). These factors, together with the small number of trials, may have limited the ability to detect a treatment effect. Established pharmacological smoking-cessation treatments also remain important comparators when evaluating the incremental clinical value of iTBS (44).

The significant subgroup difference supports caution against assuming a uniform effect of iTBS across SUDs. It may reflect true substance-related differences, but it may also arise from between-study variation in baseline severity, duration of use, stimulation dose, cortical target, craving scale, and study quality. Future trials should use standardized outcome definitions, adequately concealed randomization, credible sham procedures, and prospectively specified substance-specific protocols.

The restriction to prefrontal stimulation improved clinical comparability and was supported by the central role of prefrontal systems in executive control and cue regulation. Even within this scope, however, the left dorsolateral, right dorsolateral, and medial prefrontal targets are not functionally interchangeable. Neuroimaging-guided targeting and individualization according to circuit dysfunction may be useful directions for future research (14), but current evidence is insufficient to recommend one prefrontal target over another.

The substantial heterogeneity in the overall craving analysis is therefore clinically meaningful rather than a purely statistical issue. The pooled SMD provides an average across heterogeneous disorders and protocols and should not be used as a single expected effect for all patients with SUDs. The lower heterogeneity within the methamphetamine and nicotine subgroups suggests that substance type explained part, but not necessarily all, of the variability.

From a clinical perspective, iTBS has a practical advantage over conventional high-frequency rTMS because a standard iTBS session can be delivered within a much shorter time, which may reduce treatment burden and improve feasibility in settings where repeated visits are difficult (13). However, the present review did not directly compare iTBS with conventional high-frequency rTMS, and treatment efficiency should be distinguished from therapeutic effectiveness. The present findings support further evaluation of iTBS as an adjunctive intervention, particularly for methamphetamine use disorder, but do not justify replacing established psychosocial or pharmacological care (44, 45).

### 4.2 Abstinence, clinical implications, and durability

The abstinence analysis requires a different interpretation from the craving analysis. The three contributing trials involved nicotine or alcohol use disorder, and no methamphetamine trial contributed to this outcome. The pooled OR therefore cannot be used to infer that iTBS improves abstinence specifically in methamphetamine use disorder or across all SUDs.

Abstinence is clinically important, but its definition, verification method, and assessment time point varied across trials. In addition, most follow-up periods were no longer than 4 weeks. Consequently, the observed association may reflect an early treatment effect and does not establish durable abstinence, prevention of relapse, or a reduced need for maintenance sessions.

iTBS may ultimately be most useful as one component of multimodal treatment. Prefrontal stimulation could potentially facilitate cognitive control or learning processes relevant to behavioral therapy, but this possibility was not directly tested in the included trials and remains speculative (38, 46, 47). Trials designed to evaluate iTBS combined with standardized behavioral interventions are needed before synergistic effects can be claimed.

The present evidence also does not define an optimal dose. Treatment schedules ranged from 10 to 28 sessions, intensities varied from 80 to 110% of resting motor threshold, and targets differed. Dose–response studies and longer follow-up are needed to determine whether accelerated schedules, maintenance sessions, or individualized targeting improve clinical durability without compromising tolerability (13, 47, 48).

The imbalance of the evidence should be emphasized. Methamphetamine craving was represented by five trials and 311 participants, whereas nicotine craving was represented by three trials and 201 participants; alcohol use disorder was represented by a single 44-participant trial that contributed only to abstinence. In addition, more than half of the included trials were conducted in China and focused on methamphetamine use disorder, often in structured rehabilitation or residential treatment contexts. Cultural attitudes toward treatment, patterns of substance use, background psychosocial care, and health-system organization may differ across countries. The precision and apparent consistency of the methamphetamine estimate should therefore not be generalized to less studied substances or to settings outside those represented in the current evidence base.

Taken together, the results support a substance-specific research framework. For methamphetamine use disorder, the signal is sufficiently consistent to justify larger confirmatory trials. For nicotine and alcohol use disorders, the priority is to establish whether target selection, treatment dose, outcome timing, and integration with standard care can produce clinically meaningful and durable effects.

These findings should be interpreted as evidence of differential associations across the available trials, not as proof that a particular substance determines response. Direct comparative trials and individual-participant data meta-analyses will be required to distinguish substance-specific effects from differences in study design and participant characteristics.

Publication bias should also be considered qualitatively. Because each quantitative analysis included fewer than 10 studies, formal small-study-effect tests would have had low power and were not performed. However, the small number of available trials and the predominance of positive findings in methamphetamine studies mean that unpublished negative or inconclusive studies could alter the apparent magnitude and consistency of the methamphetamine-specific estimate.

### 4.3 Limitations

Several limitations should be considered. First, only nine trials were available, and the evidence was unevenly distributed across substances. Second, the overall craving analysis showed substantial heterogeneity, while differences in stimulation target, intensity, session number, sham procedure, concomitant treatment, and craving scales limited comparability. Third, most follow-up periods were 4 weeks or shorter, so long-term efficacy, relapse prevention, and the need for maintenance stimulation remain uncertain. Fourth, allocation concealment and prespecified reporting were often insufficiently described. Fifth, because fewer than 10 trials were included in each analysis, formal tests for publication bias were not performed; qualitatively, publication bias and small-study effects cannot be excluded, particularly given the small number of available trials and the predominance of positive findings in methamphetamine studies. Sixth, geographical and cultural generalizability is limited because more than half of the included trials were conducted in China and focused on methamphetamine use disorder. Finally, PROSPERO registration occurred after the review had commenced; this timing has now been reported transparently and should be considered when evaluating protocol adherence.

## 5 Conclusion

Prefrontal iTBS may reduce craving in methamphetamine use disorder, but current evidence is insufficient to conclude that iTBS is efficacious across SUDs more broadly. Current evidence does not demonstrate a benefit for nicotine craving. A preliminary pooled association with abstinence was observed across three nicotine or alcohol trials, but the available data are insufficient to establish durable abstinence or relapse prevention. Larger, rigorously designed, substance-specific trials with standardized protocols and longer follow-up are required before iTBS can be recommended as a routine evidence-based treatment for SUDs.

## Data Availability

The original contributions presented in the study are included in the article/Supplementary material, further inquiries can be directed to the corresponding author.

## Appendix 1

Complete database search strategies.

The search was performed from database inception to March 17, 2026. The following strategies were adapted to the syntax of each database. No restriction by substance type was applied beyond the search terms. The search was restricted to peer-reviewed English-language reports.

PubMed.

((“intermittent theta burst”[Title/Abstract] OR “intermittent theta-burst”[Title/Abstract] OR “theta burst stimulation”[Title/Abstract] OR “theta-burst stimulation”[Title/Abstract] OR iTBS[Title/Abstract] OR TBS[Title/Abstract]) AND (“Substance-Related Disorders”[Mesh] OR addiction[Title/Abstract] OR “substance use disorder”[Title/Abstract] OR “substance use disorders”[Title/Abstract] OR methamphetamine[Title/Abstract] OR nicotine[Title/Abstract] OR tobacco[Title/Abstract] OR smoking[Title/Abstract] OR alcohol[Title/Abstract] OR cocaine[Title/Abstract] OR opioid[Title/Abstract] OR opioids[Title/Abstract] OR cannabis[Title/Abstract]) AND (randomized[Title/Abstract] OR randomised[Title/Abstract] OR sham[Title/Abstract] OR placebo[Title/Abstract] OR trial[Title/Abstract] OR controlled[Title/Abstract]))

Embase.

(“intermittent theta burst”:ti,ab,kw OR “intermittent theta-burst”:ti,ab,kw OR “theta burst stimulation”:ti,ab,kw OR “theta-burst stimulation”:ti,ab,kw OR itbs:ti,ab,kw OR tbs.:ti,ab,kw) AND (“substance use disorder”/exp. OR addiction:ti,ab,kw OR “substance use disorder”:ti,ab,kw OR “substance use disorders”:ti,ab,kw OR methamphetamine:ti,ab,kw OR nicotine:ti,ab,kw OR tobacco:ti,ab,kw OR smoking:ti,ab,kw OR alcohol:ti,ab,kw OR cocaine:ti,ab,kw OR opioid*:ti,ab,kw OR cannabis:ti,ab,kw) AND (random*:ti,ab,kw OR sham:ti,ab,kw OR placebo:ti,ab,kw OR trial:ti,ab,kw OR controlled:ti,ab,kw)

Cochrane Library.

(“intermittent theta burst” OR “intermittent theta-burst” OR “theta burst stimulation” OR “theta-burst stimulation” OR iTBS OR TBS) in Title Abstract Keyword AND (addiction OR “substance use disorder” OR “substance use disorders” OR methamphetamine OR nicotine OR tobacco OR smoking OR alcohol OR cocaine OR opioid OR opioids OR cannabis) in Title Abstract Keyword AND (randomized OR randomised OR sham OR placebo OR trial OR controlled) in Title Abstract Keyword.

Web of Science Core Collection.

TS = ((“intermittent theta burst” OR “intermittent theta-burst” OR “theta burst stimulation” OR “theta-burst stimulation” OR iTBS OR TBS) AND (addiction OR “substance use disorder” OR “substance use disorders” OR methamphetamine OR nicotine OR tobacco OR smoking OR alcohol OR cocaine OR opioid* OR cannabis) AND (random* OR sham OR placebo OR trial OR controlled)).

PEDro. Because PEDro has a simplified search interface, the following searches were run separately and results were combined before deduplication: “intermittent theta burst addiction”; “intermittent theta-burst addiction”; “theta burst stimulation addiction”; “iTBS substance use”; “theta burst stimulation methamphetamine”; “theta burst stimulation nicotine”; “theta burst stimulation smoking”; and “theta burst stimulation alcohol.”
